# Respiratory Motion Mitigation and Repeatability of Two Diffusion-Weighted MRI Methods Applied to a Murine Model of Spontaneous Pancreatic Cancer

**DOI:** 10.3390/tomography7010007

**Published:** 2021-02-20

**Authors:** Jianbo Cao, Hee Kwon Song, Hanwen Yang, Victor Castillo, Jinbo Chen, Cynthia Clendenin, Mark Rosen, Rong Zhou, Stephen Pickup

**Affiliations:** 1 Department of Radiology, University of Pennsylvania, Philadelphia, PA 19104, USA; caojianbo272@gmail.com (J.C.); HeeKwon.Song@pennmedicine.upenn.edu (H.K.S.); hanweny@sas.upenn.edu (H.Y.); victorca@seas.upenn.edu (V.C.); Mark.Rosen@pennmedicine.upenn.edu (M.R.); pickup@pennmedicine.upenn.edu (S.P.); 2 Department of Biostatistics, Epidemiology and Informatics, University of Pennsylvania, Philadelphia, PA 19104, USA; jinboche@pennmedicine.upenn.edu; 3 Pancreatic Cancer Research Center, University of Pennsylvania, Philadelphia, PA 19104, USA; cclenden@pennmedicine.upenn.edu; 4 Abramson Cancer Center, University of Pennsylvania, Philadelphia, PA 19104, USA

**Keywords:** radial k-space sampling, diffusion-weighted MRI, apparent diffusion coefficient, repeatability, pancreatic ductal adenocarcinoma

## Abstract

Respiratory motion and increased susceptibility effects at high magnetic fields pose challenges for quantitative diffusion-weighted MRI (DWI) of a mouse abdomen on preclinical MRI systems. We demonstrate the first application of radial k-space-sampled (RAD) DWI of a mouse abdomen using a genetically engineered mouse model of pancreatic ductal adenocarcinoma (PDAC) on a 4.7 T preclinical scanner equipped with moderate gradient capability. RAD DWI was compared with the echo-planar imaging (EPI)-based DWI method with similar voxel volumes and acquisition times over a wide range of *b*-values (0.64, 535, 1071, 1478, and 2141 mm^2^/s). The repeatability metrics are assessed in a rigorous test–retest study (*n* = 10 for each DWI protocol). The four-shot EPI DWI protocol leads to higher signal-to-noise ratio (SNR) in diffusion-weighted images with persisting ghosting artifacts, whereas the RAD DWI protocol produces relatively artifact-free images over all *b*-values examined. Despite different degrees of motion mitigation, both RAD DWI and EPI DWI allow parametric maps of apparent diffusion coefficients (ADC) to be produced, and the ADC of the PDAC tumor estimated by the two methods are 1.3 ± 0.24 and 1.5 ± 0.28 × 10^−3^ mm^2^/s, respectively (*p* = 0.075, *n* = 10), and those of a water phantom are 3.2 ± 0.29 and 2.8 ± 0.15 × 10^−3^ mm^2^/s, respectively (*p* = 0.001, *n* = 10). Bland-Altman plots and probability density function reveal good repeatability for both protocols, whose repeatability metrics do not differ significantly. In conclusion, RAD DWI enables a more effective respiratory motion mitigation but lower SNR, while the performance of EPI DWI is expected to improve with more advanced gradient hardware.

## 1. Introduction

Genetically engineered mouse (GEM) models of pancreatic ductal adenocarcinoma (PDAC), in which the cancer arises spontaneously in pancreas, [1,2] recapitulate key features of the human disease, including a dense extracellular matrix (stroma), which represents a unique tumor microenvironment in PDAC. These models have become a major tool for the development of biomarkers and treatment pipelines [3,4,5,6,7,8]. Diffusion-weighted MR imaging (DWI) provides quantitative metrics related to the translational mobility of water hindered by microstructures present in biological tissues [9,10]. Due to its sensitivity to changes in the tumor microenvironment and cell density, DWI has been frequently employed in oncology for the diagnosis and detection of treatment responses [11,12,13,14]. Since DWI pulse sequences are sensitized to motion on the micrometer scale, macroscopic (millimeter) scale movement such as respiratory motion can introduce artefacts, e.g., ghosting, resulting in errors in the quantitative measurement of water diffusion in tissues. Located in the retroperitoneum, the pancreas is presumably less susceptible to respiratory motion than other upper abdominal organs, such as the liver. However, 4D CT and 4D MRI studies have shown that the amplitude of pancreas movement during breathing is comparable to that of the liver and lungs [15,16,17]. For example, Ferris et al. demonstrated 5–7-mm root-mean-square displacement of the human pancreas [18]. To minimize the respiratory motion artifacts, clinical DWI employs rapid acquisition protocols, such as single-shot echo-planar imaging (EPI) following diffusion weighting preparation [19]. However, unique challenges arise in the reverse translation of clinical EPI DWI techniques to the preclinical instrument: (1) a higher magnetic field strength leads to proportionally greater magnetic susceptibility effects [20], (2) faster respiration rates of mice exacerbate motion interference, (3) less advanced gradient hardware on preclinical scanners severely limits the echo train length that can be employed in EPI acquisitions, and (4) the small dimensions of the mouse limit the number of elements in array coils [21]; hence, the acceleration factor is usually less than two. These factors combined lead to the requirement of using the multi-shot EPI method in DWI studies or suboptimal image quality in single-shot EPI DWI of mouse abdomens on preclinical scanners [22,23,24,25].

Physical restrictions of respiratory motion, such as casting live mice in alginate-molds [26] or applying a rigid structure [27], appear to be effective for suppressing motion artifacts. However, concerns of respiratory distress in compromised mice may limit their application. New acquisition methods, including spatiotemporal encoding (SPEN) [28], motion-tolerant overlapping-echo detachment (DM-OLED) [29], and the use of intermolecular dipole–dipole interactions [30] have been proposed to minimize the sensitivity to motion, but in each case, motion robustness comes at the expense of other image quality factors, such as reduced spatial resolution, limited *b*-values, or low SNR. In comparison, radial k-space sampling (RAD) is inherently insensitive to motion due to the over-sampling of the k-space center [31] and does not trade image quality for motion robustness. On clinical scanners, dynamic contrast-enhanced (DCE)-MRI and DWI studies of patients with liver, lung, breast, and prostate cancers benefit from radial k-space sampling for the effective mitigation of respiratory motion interference [32,33,34,35].

RAD DWI has not been investigated in murine models of orthotopically implanted or spontaneous pancreatic cancer or in mouse abdominal organs in general. We report here the first such study in a GEM model of pancreatic cancer in comparison with a multi-shot EPI DWI protocol. We compared the SNR and image quality of the two protocols, i.e., for producing artifact-free diffusion-weighted images of pancreatic cancer in situ and their accuracy in estimating the diffusion coefficients of phantoms. In addition, we performed a rigorous test–retest study as recommended by the quantitative imaging biomarkers alliance (QIBA) [36] and reported the repeatability metrics of the apparent diffusion coefficient (ADC). These data provided reference ADC values of the PDAC in a GEM model and the repeatability metrics of two translational protocols suitable for the DWI of abdominal organs/cancers in freely breathing mice.

## 2. Methodology

### 2.1. GEM Model of Pancreatic Cancer

All animal handling procedures were reviewed and approved by the local Institutional Animal Care and Use Committee. We employed a GEM model of PDAC, referred to as KPC mice, in which *Kras* (“K”) and *p53* (“P”) mutations were introduced to the pancreatic epithelium via *Cre* recombinase (“C”) [1]. The model was bred at the Pancreatic Cancer Mouse Hospital of the Abramson Cancer Center of our institute. KPC mice spontaneously developed premalignant Pancreatic Intraepithelial Neoplastic (“PanIN”) lesions at 7–10 weeks of age, leading to invasive PDAC at 17–19 weeks with high penetrance and reproducible kinetics. Screening for tumors was done via weekly abdominal palpations starting at 11 weeks of age, followed by ultrasound examination (Vevo 2100, VisualSonics, Toronto, ON, Canada) to estimate the tumor sizes. Mice with confirmed tumor masses (both sexes, 18–25 weeks old) were enrolled in the MRI study.

### 2.2. In Vivo MRI and Test-Retest Study

MRI was performed on a 4.7 T horizontal bore system (Agilent, Palo Alto, CA, USA) equipped with a 12-cm, 25 gauss/cm gradient coil with a maximum slew rate of 11.5 T/cm/s. Animals were anesthetized via free breathing of 1.5–3% isoflurane in oxygen administered through a nose cone. Probes for monitoring the respiration rate and rectal temperature (SAI Inc., Stonybrook, NY, USA) were placed, and each mouse was positioned on a bed that was mounted into a 35 × 40 mm (ID × length) quadrature birdcage RF coil (M2M Imaging, Cleveland, OH, USA). Core body temperature was maintained at 37 ± 0.2 °C during MRI exams by directing a regulated air source into the bore. Respiration rate was maintained in the range of 80–100 breaths/min during imaging by adjustment of the isoflurane level. Two sealed 5-mm NMR tubes (Wilmad Glass, Vineland, NJ, USA) containing water and pure 1-butanol, respectively, were placed underneath the mice in the slotted mouse beds.

Following scanner calibrations and the generation of scout images, contiguous, axial, *T*_2_ weighted images spanning the tumor were acquired using a fast spin-echo protocol: effective echo time (*TE*) = 45 ms, repetition time (*TR*) = 1.5 s, slice thickness = 0.5 mm, field of view (FOV) = 32 × 32 mm^2^, slices = 12–20, matrix = 128 × 128, averages = 2, bandwidth = 50 kHz, echo train length = 8, and echo spacing = 15 ms. The radially sampled diffusion-weighted spin-echo protocol (DW-SE-RAD) and diffusion-weighted spin-echo 4-shot EPI (DW-SE-EPI) protocol, both with prospective respiratory gating, were then applied. For **DW-SE-RAD**: *TE* = 30 ms, effective *TR* = 2 to 3 respiratory periods (0.6–0.75 s per respiratory period) based on the number of slices expanding the entire tumor, one radial view (spanning the diameter of the k-space) acquired per TR, FOV = 32 × 32 mm^2^, slice thickness = 0.5 mm, slices = 12–18, matrix = 64 read × 101 views, averages = 1, bandwidth = 50 kHz, and total acquisition time = 20–38 min, depending on the respiratory rate and effective TR. For **DW-SE-EPI:** 4-shot EPI with an interleaved phase encoding the trajectory for each shot, *TE* = 26 ms, *TR* = 4 respiration periods, same FOV, slice thickness = 1.5 mm, slices = 4–6, matrix = 128 × 128, averages = 16, bandwidth = 250 kHz, and total acquisition time = 20–35 min. The slice thickness and *TR* employed for EPI represent the minimal values allowed by the gradient hardware. The remaining acquisition parameters were designed to keep the voxel volumes and total acquisition times of the two protocols similar to facilitate a fair comparison. Both DWI protocols employed identical diffusion times (Δ = 14.4 ms); diffusion gradient durations (*δ* = 9 ms); and five *b*-values (0.64, 535, 1071, 1478, and 2141 mm^2^/s). The minimum *b*-value was selected to minimize the contributions from the intravoxel incoherent motion (micro perfusion) [37]. All diffusion gradients were oriented along the diagonal, *g_diff_* = (1:1:1) to minimize the contributions from intermolecular dipole–dipole interactions [38] to the observed signals. Images were acquired with both positive and negative (-*g_diff_*) diffusion gradients, resulting in a total of 10 images per slice location. Fat saturation was not applied in either protocol to avoid the suppression of 1-butanol ^1^H signals arising in the aliphatic region (0.9–1.5 ppm) of its proton spectrum.

In clinical studies, the RAD and EPI acquisition methods have been shown to be robust to respiration motion, even in the absence of respiratory gating [32]. The need for such gating was assessed in two tumor-bearing mice in which respiratory-gated and non-gated data were acquired sequentially from each mouse. In the non-gated scans, the *TR* was set to mimic the effective *TR* used in the gated scans (e.g., 2 to 3 respiratory periods for RAD).

In the test–retest study, eight mice received DW-SE-RAD and DW-SE-EPI applied sequentially to each mouse during the “test” and “retest” scans. After the test scan, the mice were recovered from anesthesia and returned to the cage to have free access to water and food for 2–4 h. All procedures were repeated during the retest scan. Two additional mice received only RAD DWI for the test and retest scans, and another two received only EPI DWI. Hence, for each DWI protocol, test and retest data were obtained from ten mice (*n* = 10).

### 2.3. Data Processing

EPI data were phase-corrected and transformed to images using the vendor software. Radial *k*-space data were zero- and first-order phase-corrected and regridded to 64 × 64 images offline via a filtered back-projection procedure. Images acquired with positive and negative diffusion gradients were combined by taking the geometric mean of the two in order to minimize the contributions of background and imaging gradients to the signal attenuation [39]. The resulting images were subject to pixel-wise three-parameter least squares fit to Equation (1) using all five *b*-values:

(1) $$S\left(b\right)=S_{0}exp\left\{-bD\right\}+A$$

where *S*_0_, *D*, and *A* are the adjustable parameters. *S*_0_ is the signal intensity in the absence of diffusion weighting, *D* is the ADC, and *A* is a constant that accounts for the noise floor and slowly diffusing water contributions to the observed signal. Maps of these parameters were generated for further analysis. All offline data processing was performed using custom codes developed in Interactive Data Language (IDL; Harris Geospatial Solutions, Broomfield, CO, USA). Regions of interest (ROI) representing the tumor, spinal muscle, and two phantoms were manually drawn by trained staff (JC and SP) using ImageJ (National Institute of Health, Bethesda, MD, USA) [40]. Mean ADC values were obtained over the entire tumor volume and for the phantom and spinal muscles over all slices containing the tumor.

### 2.4. Statistical Analysis

To determine the correlation of ADC obtained from the two DWI protocols, we first applied the generalized linear model (GLM) to the test and the retest data separately to compute the ADC correlation coefficient (R^2^) between the two protocols. In eight mice, each received both DWI protocols in both the test and retest scans; the combined test and retest data were not suitable for a linear model, because they were not independent. To assess the correlation in the combined data, we employed the generalized estimating equations (GEE) approach to assess the linear regression using the mean ADC of each mouse estimated from the RAD protocol as the response variable and that from the EPI protocol as the independent variable in statistics software *R* version 3.6.3 (www.r-project.org) (28 May 2020) (R Foundation for Statistical Computing, Vienna, Austria).

For each protocol, Bland-Altman plots were employed to assess the bias and precision of ADC from the test–retest study. Within-subject standard deviation (*SD_ws_*) and coefficient of variation (*CV_WS_*) were calculated as follows:

(2) $${SD}_{ws}\;=\;\sqrt{\left[(\sum^{\;}\Delta D^{2})/2n\right]}$$

(3) $${CV}_{WS}\;=\;\sqrt{\left[\sum^{\;}{\left(\frac{\Delta D}{m}\right)}^{2}/2n\right]}$$

where Δ*D* is the difference between the test–retest ADC estimates for a given animal, *m* is the mean of the measurements for a given pair, *n* is the number of paired test–retest studies (*n* = 10) for each DWI protocol, and the sum is taken over *n*. The repeatability coefficient (*RC*) was calculated at a confidence level of 95% [36]:

(4) $$RC=\;1.96\;\sqrt{2SD_{ws}{}^{2}}\;=\;2.77\;\times \;SD_{ws}$$

To illustrate the ADC heterogeneity within a tumor and to compare the pixel-wise ADC distributions between the test and retest scans, plots of the probability density function (*PDF*) were made using *R* software.

## 3. Results

**Figure 1:**
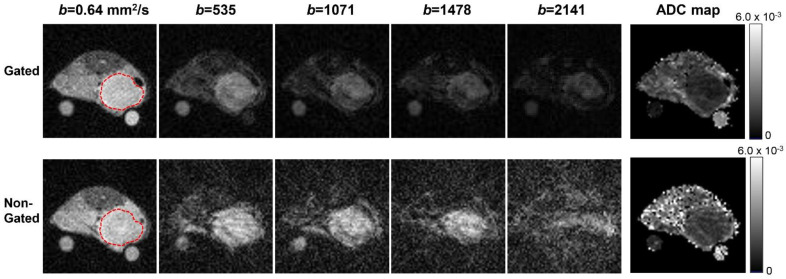
Diffusion-weighted images and the resulting apparent diffusion coefficient (ADC) maps from the radially sampled diffusion-weighted spin-echo (DW-SE-RAD) protocol with and without prospective respiratory gating. The ADC estimates of the tumor (mean ± SD from multiple slices spanning the tumor in units of × 10^−3^ mm^2^/s) are 1.1 ± 0.097 vs. 1.3 ± 0.17 (gated vs. non-gated), while estimates of the diffusion coefficient of the water phantom (in units of × 10^−3^ mm^2^/s) are 3.2 ± 0.13 vs. 4.0 ± 0.30 (gated vs. non-gated).

Images from the DW-SE-EPI protocol without prospective gating were motion-corrupted, and meaningful ADC maps could not be generated (data not shown). In comparison, although non-respiratory-gated DW-SE-RAD images exhibit increased noise and streaking artifacts compared to their gated counterparts (Figure 1), both allow the construction of good quality ADC maps, confirming the motion-insensitive benefit of radial k-space sampling. Higher levels of artifacts and potential signal loss from motion during the diffusion time seem to lead to faster apparent signal decay and increased ADC (1.1 ± 0.097 vs. 1.3 ± 0.17 × 10^−3^ mm^2^/s in tumors and 3.2 ± 0.13 vs. 4.0 ± 0.30 in the water phantom for gated and non-gated, respectively). These results demonstrate that both RAD and EPI protocols would benefit from prospective gating, which was applied in the subsequent studies presented below.

**Figure 2:**
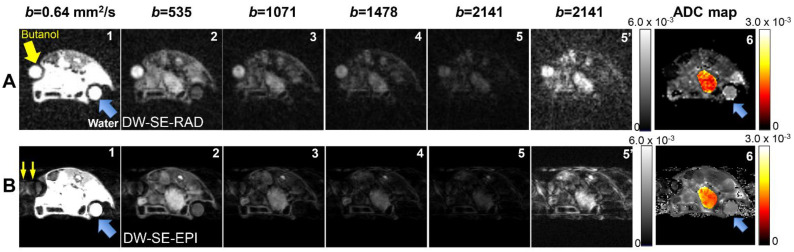
Representative diffusion-weighted images and ADC maps obtained from the two diffusion-weighted MRI (DWI) protocols. Rows **A** and **B** are diffusion-weighted images at similar slice positions generated by the DW-SE-RAD and diffusion-weighted spin-echo 4-shot echo-planar imaging (DW-SE-EPI) protocol, respectively. For each row, the first five images (**1–5**) are windowed to highlight the decay due to diffusion weighting, while frames (**A5’**,**B5’**) are windowed to optimally display (**A5,B5**), respectively. (**A6**,**B6**) are ADC maps (mm^2^/s) displayed in color overlay for the tumor and in grayscale for other tissues and phantoms. Blue arrows point to the water phantom, while yellow arrows to the 1-butanol phantom. Due to its low diffusion coefficient (~0.44 × 10^−3^ mm^2^/s), 1-butanol appears dark in the gray-scaled ADC map (**A6**) produced from the DW-SE-RAD protocol.

Representative images generated by the two DWI protocols with prospective gating are depicted in Figure 2. In rows *A* (RAD) and B (EPI), frames 1–5 are windowed to provide the optimal visualization of the attenuation due to the different levels of diffusion weighting. The signal attenuation with increasing *b*-values is comparable between the two protocols. The anatomy and tumor are clearly visualized in both datasets. At similar total acquisition times, the EPI protocol has a higher SNR (in the lowest *b*-value image) of 150 for the water phantom compared to 15 with the RAD protocol. This is due, in large part, to the higher efficiency and longer *TR* of the EPI acquisition protocol. The average SNRs in tumors are 119 and 20 for EPI and RAD, respectively, a smaller relative difference than that of water, likely due to the shorter T1 in tumors. Geometric distortion in the EPI images was minimal due to optimal shimming and the relatively short echo train employed. Panels Figure 2(A5’,B5’) are the same images as Figure 2(A5,B5), respectively but are windowed to highlight the image artifacts. In the EPI images, ghosting is evident in the phase-encoding direction (left to right) at all *b*-values and has a comparable magnitude to the desired signal in the high *b*-value images. Such ghosting is likely due to shot-to-shot phase errors [41] induced by respiration motion. In contrast, no coherent artifacts are evident in the DW-SE-RAD images. The corresponding ADC maps are shown in Figure 2(A6,B6) in color for the tumor and grayscale for the other tissues and phantoms.

The signal from the water phantom (blue arrows) drops to noise levels for the *b*-values over 1000 mm^2^/s for both protocols, consistent with the fact that the diffusion coefficient of free water is much higher than those of most biological tissues; therefore, a 1-butanol phantom was included to better match the tissue ADC values [42]. While, in DW-SE-RAD images, the signal from 1-butanol (yellow arrow) remains above the noise at the largest *b*-value, it is corrupted at all b-values in the DW-SE-EPI images due to the off-resonance effects. The ^1^H spectrum of 1-butanol consists of four resonances at 3.51, 1.56, 1,25, and 0.8 ppm, with most of the signal centered around 1.56 ppm. The phase correction scheme employed for the generation of EPI images only compensates for the dominant water peak (4.7 ppm), leading to the observed signal corruption.

Diffusion coefficient estimates for the water phantom had good reproducibility for both protocols with mean ± standard deviations of 3.2 ± 0.29 and 2.8 ± 0.15 × 10^−3^ mm^2^/s for the DW-SE-RAD and DW-SE-EPI protocols, respectively, compared to the literature value of 3.20 × 10^−3^ mm^2^/s at 37 °C [43]. However, the mean difference was found to be significant (*p* = 0.001, Table 1). This underestimation with EPI was also observed in phantom-only scans in which no mouse was loaded, excluding the possibility that respiratory motion artifacts may be the cause. Since we did not measure the temperature of the water phantom directly, we cannot be certain that they were at 37 °C, even if the core temperatures of the mice were maintained at that temperature. This could lead to uncertainties in the actual ADC value of the water phantom. The diffusion coefficient of the 1-butanol phantom estimated by the RAD DWI protocol was 0.44 ± 0.22 × 10^−3^ mm^2^/s, which compares favorably with the reported values (0.3–0.628 × 10^−3^ mm^2^/s at 25–30 °C) [42]; however, such an estimation was not feasible for EPI DWI due to the off-resonance effects.

**Figure 3:**
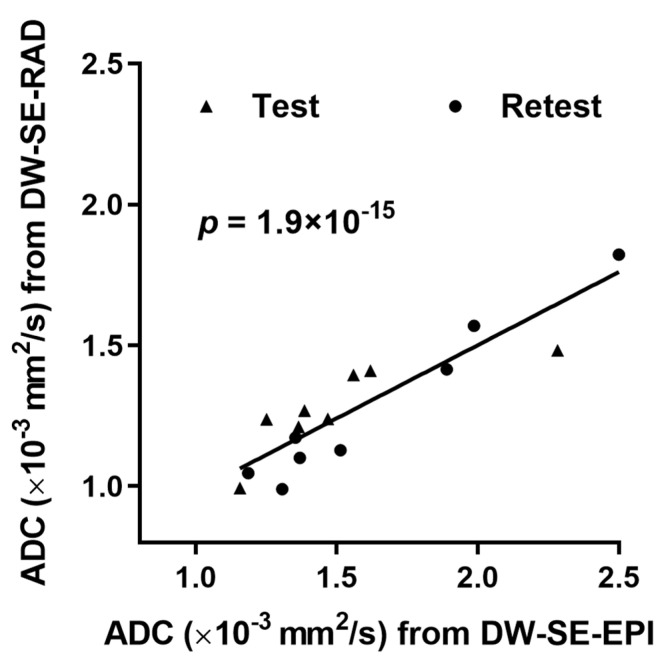
Correlation analysis of the tumor ADC values estimated by the DW-SE-RAD vs. DW-SE-EPI protocols using the generalized estimating equations approach. The analysis was applied to mice (*n* = 8) that were subjected to both DWI protocols in both the test and retest scans (detailed in Methods).

For the ADC estimates of the PDAC tumor, a strong correlation between the two protocols was obtained from the test (R^2^ = 0.67, *p* < 0.05) and the retest (R^2^ = 0.96, *p* = 0.0001) datasets, separately. When the test and retest data were combined, the GEE metrics also revealed a statistically significant correlation of the tumor ADC estimates between the DW-SE-RAD vs. DW-SE-EPI methods (*p* = 1.9 × 10^−15^) (Figure 3).

The statistical metrics generated from the test–retest study are presented in Table 1 for the water phantom, PDAC tumor, and spinal muscle. The RAD DWI protocol yields smaller *SD_ws_*, *CV_WS_*, and *RC* of the ADC estimates for both tumor and muscle, suggesting a trend of higher repeatability, although the differences are not statistically significant. For the water phantom, the trend is reversed in favor of EPI DWI. These observations suggest that a more effective motion mitigation may underlie the observed trend in the repeatability metrics.

**Figure 4:**
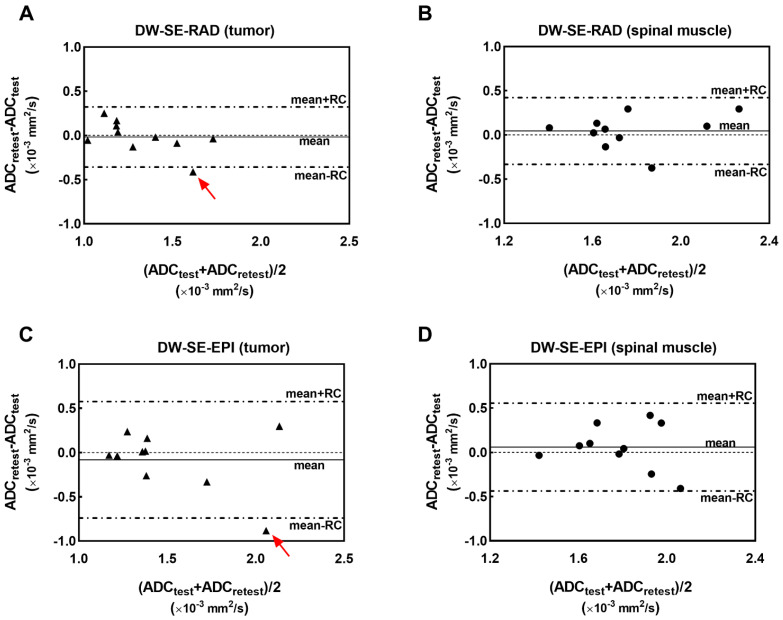
Bland-Altman plots of the tumor (**A**,**C**) and muscle (**B**,**D**) ADC measurements obtained from the test–retest experiment for each DWI protocol. The mean of ΔADC and mean *± RC* are specified in the figure, while the *RC* values are reported in Table 1. Red arrows point to the worst cases in each DWI protocol. ΔADC = ADC_test_ − ADC_retest_, *RC* is repeatability coefficient defined in Equation [4].

Bland-Altman plots of ADC values generated from the test–retest study are shown in Figure 4 for the tumor and muscle. For both protocols, the biases for both tissues are very small relative to *RC*, indicating a minimal variation in the ADC estimates in the test–retest setting. The intra-subject difference of the ADC (ΔADC, *y*-axis values) does not appear to be correlated with the magnitude of their mean (*x*-axis values) for the tissues investigated here. The distances between the lines representing the mean *± RC* in Figure 4 confirm the trend of higher repeatability provided by the RAD compared to the EPI protocol.

**Figure 5:**
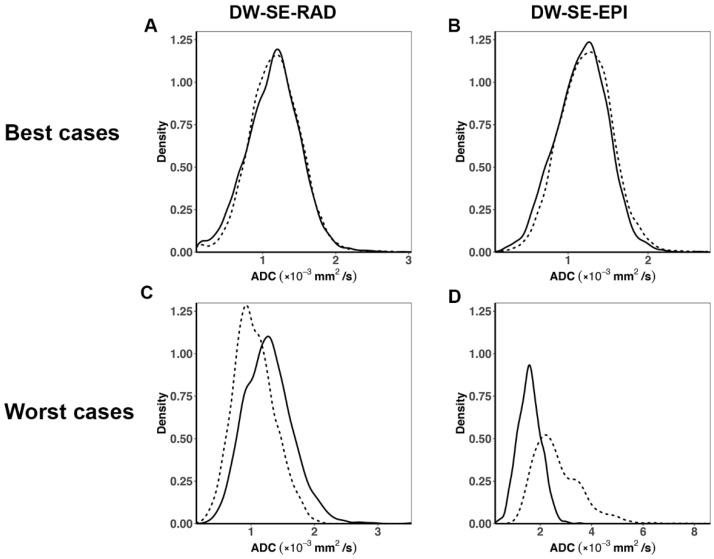
Probability density function (PDF) of the pixel-wise tumor ADC values from the best (**A**,**B**) and worst cases (**C**,**D**) in the test–retest study for the DW-SE-RAD and DW-SE-EPI protocols, respectively (solid line: test and dotted line: retest). The best and worst cases correspond to the smallest and largest ΔD values, respectively, from ten mice subjected to the test–retest study for each protocol.

Among the ten pairs of test–retest ADC values from each DWI protocol, the best and worst cases, corresponding to the smallest and largest Δ*D* (or ΔADC) values, respectively, were selected. The two worst cases are identified by red arrows in Figure 4A,C. The PDFs of the tumor ADC values for these cases are shown in Figure 5. The PDFs (analogous to histograms) reveal the heterogeneity and distribution of the ADC values within the tumor. The PDFs from test and retest scans are nearly superimposed in the best-case examples (Figure 5A,B) but are notably different in the worst-case examples, with larger differences from the EPI DWI protocol (Figure 5C,D).

## 4. Discussion

Many cancers arise from the abdominal organs, such as the liver, and colorectal cancer. While respiratory motion is known to interfere with the quantitative DWI of the mouse abdomen, this challenge can be mitigated by employing subcutaneous (subQ) models, which are produced by inoculating cancer cells under the skin of the hind limb or flank, since both locations are relatively immune to respiratory motion. Recently, there has been an increased use of GEM models, in which tumors arise from and interact with the native tissue environment and, therefore, overcome some key limitations associated with subQ models [44].

To meet the need for mitigating respiratory motions in preclinical DWI, we demonstrated the first application of radially sampled DWI of the mouse abdomen and made comparisons between the radial and EPI-based methods. While both protocols are implemented on a preclinical scanner equipped with moderate gradient hardware, DW-SE-EPI was more affected by the limited gradient capability, leading to requirements for a longer TR, thicker image slices, and multi-shot EPI acquisition. To compare RAD and EPI DWI performances under such constraints, we closely matched the voxel volume and total acquisition time. Our data showed that the radial method is superior in motion mitigation and accuracy in estimating the diffusion coefficient of the water phantom. In comparison, EPI DWI has a more favorable SNR but leads to suboptimal mitigation of motion artifacts. It is possible, though, that the relative merits of the two DWI methods could be different with improvements in the gradient and RF hardware on preclinical MRI systems. For example, a greater maximum gradient strength may enable a shorter diffusion time (Δ) and bipolar diffusion gradients, both of which appear effective in the suppression of motion artifacts in a recent EPI DWI study of murine liver cancer [22].

The lower SNR observed in the DW-SE-RAD images may potentially have been affected by the dispersion of motion-related artifacts into the background regions, since radial methods avoid coherent artifacts (e.g., ghosting) by spreading out the effects throughout the FOV, leading to apparently elevated background noise. Relatively long T1 values of tumors (and water) could also contribute to reduce the SNR in the radial acquisition due to shorter TRs than the EPI protocol. To improve image SNR, it may be possible to utilize trajectory correction schemes [45] and iterative image reconstruction methods [46] in the DW-SE-RAD protocol.

While 1-butanol may have been sufficient for radial-based methods, its off-resonance characteristics led to undesired artifacts in EPI. Our study could have been improved with the utilization of a water-based phantom with the ADC closely matching that of the tissue of interest, such as a polyvinylpyrrolidone (PVP) phantom [47].

Despite different degrees of motion mitigation, both DW-SE-RAD and DW-SE-EPI allow quantitative DWI of the mouse abdomen by producing parametric maps of the ADC. Tumor ADC values estimated from RAD vs. EPI DWI are highly correlated (Figure 3) and do not differ significantly (Table 1). The ADC values from KPC mice provide a reference for future studies and are well within the range reported from human PDAC (1.11–1.50 × 10^−3^ mm^2^/s) [48,49], suggesting similarities in the tumor architecture and microenvironment between the GEM model and human disease.

The repeatability metrics provide variability assessments for each DWI protocol. Such assessments would enable the estimation of the sample size required by each DWI method [36] and are motivated by increased consensus within the cancer imaging community to report these metrics for quantitative imaging markers [14,27,50,51,52]. Considering the highly motion-susceptible location of the pancreatic tumor in freely breathing mice, our reproducibility metrics of the DW-SE-RAD protocol (*SD_ws_* = 0.12 × 10^−3^ mm^2^/s and *CV_WS_* = 9%) compare favorably to those reported from tumors located in less motion-susceptible locations—for example, the *SD_ws_* of 0.1 × 10^−3^ mm^2^/s from breast cancer xenografts grown in the hind flank of mice [50], *SD_ws_* of 0.06 × 10^−3^ mm^2^/s, and *CV_WS_* of 7% from orthotopically implanted breast tumors in mice with restricted respiration [27].

In this work, we endeavored to keep the voxel volumes and scan times similar between the two protocols in order to facilitate performance comparisons. In this regard, it is possible that alternative choices of imaging parameters may have further improved the comparisons, such as increasing the number of segments or the number of b-values for the EPI protocol rather than simply increasing the number of averages. A greater number of EPI segments would also help reduce the gradient duty cycle. However, it is likely that much of the ghosting artifacts observed in the EPI images are due to shot-to-shot phase variations generated by motion. As such, increases in the number of segments would increase the amplitude and/or spatial frequency of phase-encoding ghosts, subsequently increasing the amount of signal overlap. Further, one of the benefits of averaging is the suppression of ghosting artifacts caused by motion. Our observation with a four-shot EPI with no averaging yielded poor-quality images (data not shown).

While exactly matching the scan parameters between the two sequences may make the comparisons fairer in some sense, one of our goals in designing these protocols was to take advantage of the best features of each method. The main motivation for employing EPI methods is the very high acquisition efficiency associated with the technique. Further reductions in the acquisition efficiency due to an increased number of segments may detract from the advantage of this technique.

One disadvantage of the radial DWI protocol is the relatively long scan time, which may make it impractical for applications that require many b-values and/or multiple diffusion gradient directions. Therefore, minimizing the total acquisition time of a DWI study is important for increasing the throughput and permitting multiple imaging markers to be obtained in the same session. To accelerate the acquisition of RAD DWI, several approaches are worth investigating, including diffusion-weighted radial fast spin-echo [53] and diffusion-weighted PROPELLER [54], which combines the desirable features of both the radial and multi-echo methods. Of note, radial sampling’s ability to tolerate under-sampling can be exploited in combination with new image reconstruction models for under-sampled radial data [55]. Consequently, such an approach may allow reducing the acquisition time while maintaining the good quality of under-sampled images.

## Figures and Tables

**Table 1:** Statistical parameters derived from the apparent diffusion coefficient (ADC) estimates in the test–retest study ^a^.

	Protocol	ADC (Mean ± SD)		Δ*D* (Mean ± SD)	*SD_ws_*	*CV_WS_*	*RC*
		Test ^b^	Retest ^b^				
Water ^c^	DW-SE-RAD	3.2 ± 0.29	3.3 ± 0.27	−0.048 ± 0.28	0.19	0.060	0.53
	DW-SE-EPI	2.8 ± 0.15	2.8 ± 0.10	0.069 ± 0.15	0.11	0.039	0.31
Muscle	DW-SE-RAD	1.8 ± 0.29	1.7 ± 0.25	0.045 ± 0.20	0.14	0.073	0.38
	DW-SE-EPI	1.8 ± 0.22	1.7 ± 0.25	0.060 ± 0.26	0.18	0.096	0.50
Tumor	DW-SE-RAD	1.3 ± 0.19	1.3 ± 0.29	−0.017 ± 0.18	0.12	0.090	0.34
	DW-SE-EPI	1.5 ± 0.32	1.5 ± 0.44	−0.082 ± 0.34	0.24	0.13	0.66

^a^: All values are reported in the unit of × 10^−3^ mm^2^/s, except for *CV_WS_* and *RC,* which are unitless. Δ*D*, *SD_ws_*, *CV_WS_*, and *RC* are defined in Equations (2)–(4). ^b^: The differences of the ADC between test and retest are not significant (*p* > 0.05). ^c^: The difference of ADC derived from radially sampled diffusion-weighted spin-echo (DW-SE-RAD) vs. diffusion-weighted spin-echo 4-shot echo-planar imaging (DW-SE-EPI) is significant for the water phantom (*p* < 0.001) but not significant for the muscle or tumor (*p* > 0.05).

## Data Availability

Image data will be shared at a data repository being built for Penn Quantitative Imaging Resource for Pancreatic Cancer (https://pennpancreaticcancerimagingresource.github.io/data.html/).

## Abbreviations

**ADC or D**	apparent diffusion coefficient
***CV_WS_***	within-subject coefficient of variation
**DWI**	diffusion-weighted MRI
**DW-SE-EPI**	diffusion-weighted 4-shot spin-echo echo planar imaging protocol
**DW-SE-RAD**	diffusion-weighted radially sampled spin-echo protocol
**GEM**	genetically engineered mouse
**PDAC**	pancreatic ductal adenocarcinoma
***PDF***	probability density function
***RC***	repeatability coefficient
***SD_ws_***	within-subject standard deviation
